# Low MMP-8/TIMP-1 reflects left ventricle impairment in takotsubo cardiomyopathy and high TIMP-1 may help to differentiate it from acute coronary syndrome

**DOI:** 10.1371/journal.pone.0173371

**Published:** 2017-03-09

**Authors:** Olavi Parkkonen, Mikko T. Nieminen, Paula Vesterinen, Taina Tervahartiala, Markus Perola, Veikko Salomaa, Pekka Jousilahti, Timo Sorsa, Pirkko J. Pussinen, Juha Sinisalo

**Affiliations:** 1 Heart and Lung Center, Helsinki University Hospital and Helsinki University, Helsinki, Finland; 2 Department of Oral and Maxillofacial Diseases, University of Helsinki and Helsinki University Hospital, Helsinki, Finland; 3 National Institute for Health and Welfare, Helsinki, Finland; 4 Division of Periodontology, Department of Dental Medicine, Karolinska Institutet, Huddinge, Sweden; University Medical Center, GERMANY

## Abstract

**Background:**

Matrix metalloproteinase 8 (MMP-8) is the most potent type-I collagen protease. Such collagen mainly constitutes the transient fibrosis in takotsubo cardiomyopathy (TTC) endomyocardial biopsies. High MMP-8 and tissue-inhibitor of matrix metalloproteinase-1 (TIMP-1) levels are implicated in acute coronary syndrome (ACS). We compared MMP-8 and TIMP-1 levels in consecutive TTC and ACS patients, and their association to TTC severity.

**Methods and results:**

In 45 acute serum samples of TTC, 2072 ACS and 1000 controls, TIMP-1 differed between ACS 146.7ng/mL (115.0–186.3) (median (interquartile range)), TTC 115.7 (94.3–137.7) and controls 80.9 (73.2–90.4), (p<0.0001). MMP-8 levels were similar between ACS and TTC. In receiver-operating characteristics analysis, TIMP-1 differentiated TTC from ACS with an area under the curve (AUC) of 0.679 (p<0.0001) surpassing troponin T (TnT) at 0.522 (p = 0.66). Compared to other differing factors (age, sex, smoking), TIMP-1 improved diagnostic specificity and sensitivity from AUC of 0.821 to 0.844 (p = 0.007). The MMP8/TIMP-1 molar ratio differentiated normal ejection fraction (EF) at 0.27 (0.13–0.51) from decreased EF<50% at 0.08 (0.05–0.20), (p = 0.04) in TTC, but not in ACS.

**Conclusions:**

Even with other differing factors considered, TIMP-1 differentiated TTC from ACS better than TnT. In TTC, the low MMP-8/TIMP-1 molar ratio may reflect decreased proteolysis and increased transient fibrosis, perhaps in part explaining the left-ventricle impairment.

## Introduction

Takotsubo cardiomyopathy (TTC), a form of acute heart failure, mimics myocardial infarction with similar electrocardiogram and cardiac enzyme findings [1]. In an acute setting, the TTC and acute coronary syndrome (ACS) are difficult to distinguish without an invasive procedure. Coronary angiograms of TTC patients show no signs of occlusive coronary artery disease, and left ventriculograms reveal a typical transient contraction abnormality which usually resolves within weeks. Previous attempts to differentiate TTC from ACS using current non-invasive methods such as ECG, cardiac enzymes, or acute-phase reactants such as C-reactive protein resulted in inadequate resolution and controversy [2,3].

Matrix metalloproteinases (MMPs) maintain extracellular matrix (ECM) under normal conditions with their ability to cleave almost all extracellular proteins such as collagens. Structurally related MMPs are secreted mainly by inflammatory cells, but also by endothelial and smooth muscle cells [4]. MMP activity is tightly controlled by tissue inhibitors of matrix metalloproteinases (TIMPs) [5,6]. Disturbance in MMP and TIMP balance may raise or lower the ECM fibrotic material and accompany inflammation. Such changes, especially in MMP-8 and TIMP-1 levels, accompany the pathogenesis of atherosclerosis and acute coronary syndrome [6–9]. In myocardial ECM, an altered MMP and TIMP balance may lead to structural and functional changes, and subsequent impairment of cardiac function as seen in heart failure and various cardiomyopathy patients [10,11].

Left ventricle presentation in TTC varies from normal ejection fraction (EF) to cardiogenic shock. Factors affecting the severity of contraction abnormality and heart failure in TTC are unknown [12]. Endomyocardial biopsies of acute TTC patients show transient ECM fibrosis [13]. Altered fibrosis due to MMP-8 and TIMP-1 imbalance may play a role in pathogenesis. Reports of small TTC patient-series showed similar MMP and TIMP profiles as in hypertension- and diastolic heart-failure patients, but include no direct comparisons to ACS [14].

We set out to compare the levels of circulating MMP-8 and TIMP-1 between acute ACS-, TTC-, and control patients. The aim was to improve non-invasive methods to differentiate ACS from TTC. We also aimed to discover whether their serum TIMP-1 and MMP-8 levels correlate with severity or variant of the contraction abnormality in TTC, and thus may play a role in the underlying pathophysiology.

## Methods

### Study populations

The study population comprised 2167 consecutive acute cardiac patients from the large COROGENE study [15]. In brief, 5294 patients were each assigned to undergo a coronary angiogram (CAG) in Helsinki University Hospital between June 2006 and March 2008. The data register is based on comprehensive patient-specific data incorporating medical records and a 2-page questionnaire with information on medical history, demographics, co-morbidities, current condition, cardiovascular risk factors, and on medications such as antihypertensive or lipid-lowering drugs. Results were included from electrocardiograms, echocardiograms, and coronary angiograms.

Acute coronary syndrome (ACS) was defined as an episode of typical chest pain for ischemia and >50% stenosis in ≥ 1 coronary artery. The electrocardiogram had to show typical ischemic changes for unstable angina pectoris (UAP), non–ST-elevation myocardial infarction (NSTEMI), or ST-elevation myocardial infarction (STEMI). The distribution of patients with ACS was as follows: with STEMI, 722 (35%), with NSTEMI, 1123 (54%), and with UAP, 226 (11%). All ACS patients were treated with standard drug regimens and procedures. The remaining 95 acute patients were further reviewed, and 45 met the following criteria for TTC: 1. acute chest pain or dyspnea or both; 2. acute ST-T changes in electrocardiogram or elevated cardiac enzymes or both; 3. absence of obstructive coronary artery disease (< 50% luminal narrowing); 4. myocarditis excluded. The 50 excluded patients had other cardiac or non-cardiac condition such as atrial fibrillation.

For TTC patients, following an angiogram, a left ventriculogram was performed with the Siemens AXIOM Artis dFC system and EF, end diastolic volume, end systolic volume, and stroke volume were measured with Siemens AXIOM Sensis software (Siemens AG, Forcheim, Germany). Involvement of apical myocardium determined the TTC subtype: the left ventricle was divided into apical 30%, mid- 35%, and basal 35% portions by adapting a method previously described for tomographic imaging of the heart [16]. Based on a clinical decision, ACS patients’ echocardiograms were performed during their hospitalization, and the EF information came from their medical records.

FINRISK is a population-based survey designed for study of the prevalence in Finland of cardiovascular disease (CVD) risk factors [17,18]. The 1997 survey included 8389 randomly selected Finnish men and women aged 25 to 74 years. Participants underwent a physical examination and completed a questionnaire regarding cardiovascular risk factors and doctor-diagnosed diseases. Controls in the present study were chosen from among the participants of the FINRISK in the same region—Helsinki and Uusimaa—as the patients in the COROGENE. After exclusion of those with prevalent or incident CVD events, prevalent or incident diabetes, and those with prevalent metabolic syndrome [19], the population comprised 1000 healthy subjects. Information on CVD events and diabetes was complemented with register data either as hospitalizations with the disease or as intake of related drugs. The incident diseases were excluded based on 13 years of follow-up. Metabolic syndrome was defined based on clinical examination, intake of drugs in connection to the diagnosis, and laboratory determinations.

The COROGENE study was approved by the ethics committee of the University of Helsinki and the FINRISK study by the ethics committee of the National Institute of Health and Welfare. The investigation conforms with the principles outlined in the Declaration of Helsinki. Written informed consent came from each of the subjects.

### Laboratory determinations

Blood samples from the Corogene population were acquired in the beginning of CAG from an arterial cannula. Citrated plasma was stored at –80°C until analyzed. High-sensitivity C-reactive protein (hsCRP) (Orion Diagnostica, Espoo, Finland) and Troponin T (TnT) (Roche Diagnostics GmbH, Basel, Switzerland) were both measured upon admission according to the laboratory standards of Helsinki University Hospital. In addition, the highest value of TnT during hospitalization was recorded.

Blood samples from the FINRISK population were collected during clinic visits. The participants were asked to fast for 4 hours and to avoid heavy meals before the blood sampling. The median fasting time was 5 (interquartile range 3–7) hours. All laboratory measurements were carried out at the Disease Risk Unit of the National Institute for Health and Welfare, Helsinki, and are described elsewhere in detail [20].

Serum MMP-8 concentrations were determined by a time-resolved immunofluorometric assay (IFMA) and TIMP-1 concentrations by enzyme-linked immunosorbent assay (ELISA; R&D Systems, Minneapolis, MN, USA) from both study populations, as described [21]. The inter-assay coefficient of variation (CV) for MMP-8 IFMA was 7.3% (28 subjects), and the detection limit was 0.08 μg/l. The inter-assay CV% for TIMP-1 ELISA was 8.2% (28 subjects), and the detection limit was 0.08 ng/ml [21].

### Statistical and data analysis

Statistical analyses were performed with SPSS v. 21.0 (SPSS Inc., Chicago, IL, USA). Significance of the differences in the characteristics between cases and controls, and within TTC patients, underwent analysis depending on the normality and size of the study groups by Student’s t-test or the Mann-Whitney U-test for the continuous variables and by Chi-square for the categorical variables. Data distribution was assessed with Kolmogorov-Smirnov and Shapiro-Wilk tests as well as visually inspected with Q-Q plots and histograms. Relationships between study covariates were tested with Pearson correlation or Spearman’s rho within cases and controls separately. To study the diagnostic ability of MMP-8, TIMP-1, and MMP-8/TIMP-1 molar ratio to distinguish cases from controls, receiver-operating characteristics (ROC) was the test used. Parameters with skewed distribution were log-transformed prior to analysis. To assess whether these determinations had value over the traditional risk factors, C-statistics were included in the analysis. A multivariate logistic model adjusted for age, sex, smoking, high-density lipoprotein (HDL), body mass index (BMI), dyslipidemia, diabetes, and hypertension served to analyse the associations between the tertiles of the concentrations and diagnoses. A p-value less than 0.05 was considered statistically significant. Depending on distribution, values are expressed either as mean ± standard deviation (SD) or median (interquartile range (IQR)). Confidence intervals (CIs) and odds ratios (ORs) are presented when applicable.

## Results

The acute patients, with ACS or TTC, were of similar age, but averaged somewhat older than controls. Male dominance was observable in the ACS group (69.4%), as opposed to female dominance in the TTC group (86.7%). In controls, the distribution was almost equal (male 44.8%). Among the cardiovascular risk factors, the controls had a lower BMI than did those with ACS or TTC. Hypertension was less frequent in controls and smoking most frequent among ACS patients. Total cholesterol and LDL cholesterol levels were highest in the control patients; the HDL cholesterol levels were lowest in the ACS group. Medication was similar for those with TTC or ACS (Table 1).

**Table 1:** Patient characteristics in case-control sample.

	ACS	TTC	Control	p-value		
	n = 2072	n = 45	n = 1000	1 and 2	1 and 3	2 and 3
Age	66 ± 12	66 ± 9	44.7 ± 13	0.810	<0.001	<0.001
BMI	27.4 ± 4.8	26.4 ± 4.8	25.0 ± 3.5	0.167	<0.001	0.063
Smoking, ever	1305 (63.0)	18 (40.0)	404 (40.4)	0.003	<0.001	0.957
Hypertension	1365 (65.9)	27 (60.0)	319 (31.9)	0.429	<0.001	<0.001
Diabetes	396 (19.1)	6 (13.3)	0 (0.0)	0.328	<0.001	<0.001
Hyperchol or statin	1187 (57.3)	23 (51.1)	690 (69.0)	0.448	<0.001	0.014
Total cholesterol (mmol/L)	4.3 ± 1.1	4.5 ± 0.8	5.4 ± 1.0	0.516	<0.001	<0.001
LDL (mmol/L)	2.4 ± 0.4	2.2 ± 0.7	3.4 ± 0.9	0.148	<0.001	<0.001
HDL (mmol/L)	1.2 ± 0.4	1.7 ± 0.6	1.5 ± 0.3	<0.001	<0.001	0.003
Triglyceride	1.2 (0.9–1.7)	1.2 (0.8–1.5)	1.0 (0.8–1.4)	0.195	<0.001	0.405
Pro-BNP	2034 (681–6013)	4478 (411–13291)	N/A	0.67	N/A	N/A
ACE-inhibitor	478 (23.1)	10 (22.2)	N/A	0.819	N/A	N/A
AT-blocker	304 (14.7)	7 (15.6)	N/A	0.917	N/A	N/A
Statin medication	750 (36.2)	14 (31.1)	12 (1.2)	0.36	<0.001	<0.001
Thrombolysis	246 (11.9)	0 (0.0)	N/A	0.014	N/A	N/A
TnT admission (μg/L)	0.3 (0.1–0.9)	0.2 (0.1–0.6)	N/A	0.657	N/A	N/A
TnT highest (μg/L)	0.84 (0.26–2.47)	0.32 (0.17–0.69)	N/A	<0.001	N/A	N/A
CK-mbm (μg/L)	8.0 (4.0–26.0)	11.0 (6.0–16.0)	N/A	0.508	N/A	N/A
hsCRP (mg/L)	5.8 (1.9–20.5)	6.5 (1.9–21.7)	0.8 (0.4–1.9)	0.886	<0.001	<0.001
TIMP-1 (ng/mL)	146.7 (115.0–186.3)	115.7 (94.3–137.7)	80.9 (73.2–90.4)	<0.001	<0.001	<0.001
MMP-8 (ng/mL)	61.5 (29.2–125.4)	58.7 (25.7–130.4)	26.3 (14.9–48.2)	0.847	<0.001	<0.001
MMP-8/TIMP-1	0.18 (0.08–0.37)	0.21 (0.08–0.46)	0.14 (0.08–0.26)	0.314	<0.001	0.012

ACE, angiotensin convertase enzyme; ACS, acute coronary syndrome; AT, angiotensin; BMI, body mass index; CK-mbm, creatine kinase mb-mass; HDL, high-density lipoprotein; hsCRP, high sentivity c-reactive protein; LDL, low-density lipoprotein; MMP-8, matrix metalloproteinase 8; Pro-BNP, beta-natriuretic propeptide; TIMP-1, tissue inhibitor of matrixmetalloproteinase; TnT, troponin T; TTC, takotsubo cardiomyopathy. Data as n (%), mean ± standard deviation or median (interquartile range). Hypercholesterolemia: total >5.0 or LDL >3.0. N/A, not available

**Fig 1:**
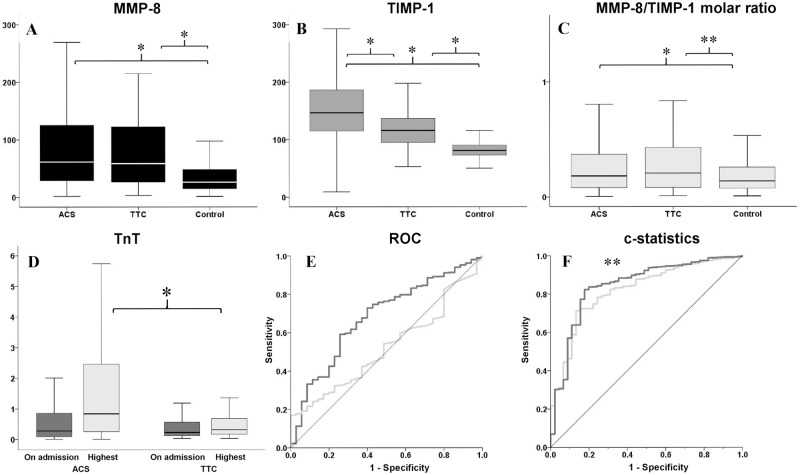
Differences in acute-phase levels of A) matrix metalloproteinase-8 (MMP-8), B) tissue inhibitor of matrix metalloproteinase-1 (TIMP-1), and C) MMP-8/TIMP-1 molar ratio of acute coronary syndrome (ACS) (n = 2072) and takotsubo cardiomyopathy (TTC) (n = 45) compared to those of controls. D) Troponin T on admission and highest value during hospitalization in ACS and TTC. E) ROC curves of TIMP-1 and troponin T in differentiation of ACS from TTC. F) ROC curves after c-statistics showing the improved ability of TIMP-1 + other differing factors (age, sex and smoking) in differentiation between TTC and ACS over other differing factors alone (Table 2). Data in box-plots are presented as medians, 25th and 75th percentiles (boxes), and 10th and 90th percentiles (whiskers).* p < 0.001; ** p = 0.01.

As expected, acute-phase protein HsCRP-, as well as cardiac enzymes TnT- and CK-MBm levels were higher in ACS and in TTC patients than in controls. Of the four, ACS and TTC patients differed only in the highest in-hospital TnT level (Table 1, Fig 1D). In TTC patients, hsCRP correlated with MMP-8 (r = 0.4; p = 0.006) and TIMP-1 (r = 0.3; p = 0.03), but not with TnT. In ACS patients, MMP-8 and TIMP-1 correlated with hsCRP (both r = 0.3; p < 0.001). Moreover, ACS patients’ TnT showed a weak correlation with MMP-8 and MMP-8/TIMP-1 molar ratio (r < 0.16; p < 0.001).

TTC and ACS patients had similar MMP-8 levels, which were higher than those of the controls. TIMP-1 levels, however, were highest in ACS patients, followed by the TTC- and control levels (p < 0.0001, for both) (Table 1, Fig 1A and 1B). When patients were divided into tertiles according to serum levels of MMP-8, TIMP-1, and MMP-8/TIMP-1 molar ratio respectively, the TIMP-1 was associated with ACS over TTC, as the 3rd vs 1st tertile OR 4.52 (1.86–11.00; p < 0.002), but MMP-8 and MMP-8/TIMP-1 molar ratio did not. (S1 Table). The association of TIMP-1 with ACS remained statistically significant in multivariate analysis (Table 2).

**Table 2:** Receiver operating characteristics, c-statistics and multivariate analysis in ACS and control compared to TTC.

	ACS	Control
	Area under the curve (95% confidence interval)	
ROC		
MMP-8	0.513 (0.423–0.603)	0.302 (0.216–0.387)*
TIMP-1	0.679 (0.606–0.753)*	0.149 (0.075–0.222)*
MMP-8/TIMP-1 ratio	0.467 (0.378–0.555)	0.413 (0.317–0.509)*
TnT admission	0.522 (0.442–0.602)	N/A
C-statistics		
Basic^†^	0.821 (0.764–0.879)*	0.075 (0.041–0.109)*
Basic + MMP-8	0.820 (0.761–0.880)*	0.061 (0.030–0.091)*
Basic + TIMP-1	0.844 (0.783–0.906)*	0.050 (0.020–0.079)*
Basic + MMP-8/TIMP-1 ratio	0.826 (0.767–0.885)*	0.064 (0.033–0.096)*
	Odds ratios (95% confidence interval)	
Multivariate analysis		
MMP-8 and Age, sex, smoking	0.999 (0.996–1.002)	1.009 (1.004–1.013)*
TIMP-1 and Age, sex, smoking	1.012 (1.005–1.018)**	1.053 (1.034–1.072)*
MMP-8/TIMP-1 and Age, sex, smoking	0.759 (0.506–1.138)	6.159 (2.748–13.803)*
MMP-8 and dyslipidemia, hypertension, diabetes^†^	0.999 (0.996–1.002)	1.008 (1.005–1.012)*
TIMP-1 and dyslipidemia, hypertension, diabetes	1.011 (1.005–1.018)**	1.069 (1.052–1.086)*
MMP-8 and age, sex, smoking, HDL	0.997 (0.994–1.000)	1.010 (1.005–1.015)*
TIMP-1 and age, sex, smoking, HDL	1.009 (1.001–1.017)***	1.057 (1.035–1.080)*
MMP-8/TIMP-1 and age, sex, smoking, HDL	0.667 (0.434–1.026)***	8.694 (3.608–20.951)*

ACS, acute coronary syndrome; HDL, high-density lipoprotein; LDL, low-density lipoprotein; MMP-8, matrix metalloproteinase; ROC, receiver operating characteristics; TIMP-1, tissue inhibitor of matrix metalloproteinase; TnT, troponin T; TTC, takotsubo cardiomyopathy.

^†^ Basic: age, sex, smoking

* p < 0.0001

** p < 0.01

*** p < 0.05

The sensitivity and specificity of the TIMP-1 in differentiation of ACS from TTC in ROC analysis showed an area under the curve (AUC) of 0.679 (95% CI 0.606–0.753; p < 0.0001) (Fig 1E). In the c-statistics, TIMP-1 raised the sensitivity and specificity over the other significant differences (age, sex, and smoking) (p < 0.01) improving the AUC from 0.821 (95% CI 0.764–0.879; p < 0.0001) to 0.844 (95% CI 0.783–0.906; p < 0.0001) (Fig 1F).

The MMP-8 and MMP-8/TIMP-1 molar ratio also produced significant results (p < 0.0001), but AUC’s were similar to those comprising age, sex, and smoking (Table 2). Controls were differentiated from TTC clearly by MMP-8, TIMP-1 (p < 0.0001) and MMP-8/TIMP-1 molar ratio (p < 0.05).

**Fig 2:**
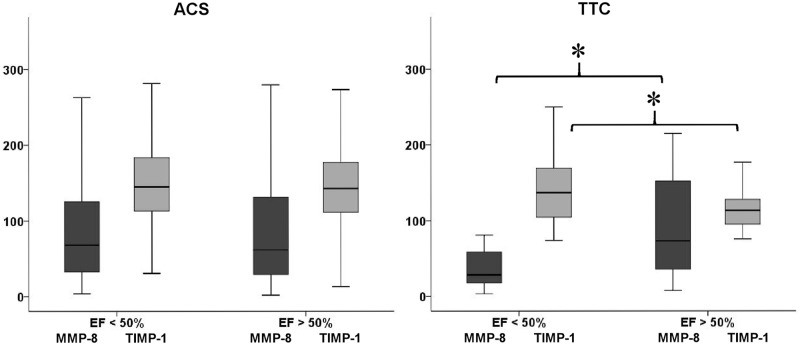
In acute coronary syndrome and Takotsubo Cardiomyopathy (TTC) patients, Matrix Metalloproteinase-8 (MMP-8) and Tissue Inhibitor of Matrix Metalloproteinase-1 (TIMP-1) levels stratified by Ejection Fraction (EF). In TTC, MMP-8 and TIMP-1 levels also reflect ventricular impairment. Data in box-plots are presented as medians, 25th and 75th percentiles (boxes), and 10th and 90th percentiles (whiskers). * p = 0.01.

Median EF in TTC patients was 53% (IQR 35–62), and 17 patients had low EF < 50%. Such patients had low MMP-8 levels, but high TIMP-1 levels compared to TTC patients with normal EF (S2 Table, Fig 2). EF correlated with MMP-8/TIMP-1 molar ratio (r = 0.358, p = 0.02), but not separately with MMP-8 or TIMP-1. In TTC, hsCRP correlated with MMP-8 and TIMP-1 (r > 0.3; p < 0.05). Left ventricular volumes (end-diastolic, end-systolic, or stroke volume) failed to correlate with MMP-8, TIMP-1, or MMP-8/TIMP-1 molar ratio. In ACS patients, neither MMP-8 nor TIMP-1 was related to EF (Fig 2). TTC patients with EF < 50% had similar TIMP-1, but lower MMP-8 levels than ACS patients. Normal EF > 50% TTC patients had lower TIMP-1 and similar MMP-8 levels than those with ACS (S2 Table, Fig 2).

Apical TTC patients had lower EF and higher cholesterol levels than did those with mid-ventricular TTC, but showed no differences in their cardiac enzymes, acute phase reactants, MMP-8, TIMP-1, or their MMP-8/TIMP-1 molar ratios (S3 Table).

## Discussion

We analysed levels of the most potent collagen type-I degrading protease, MMP-8 [22], and of its inhibitor TIMP-1 levels in acute-phase TTC and ACS patients as well as in healthy controls. To discover whether these levels could differentiate TTC patients from ACS patients, we showed that TIMP-1 level differed significantly between these study groups. Moreover, TIMP-1 could segregate TTC from ACS better than TnT could, even with other differing factors (age, sex, and smoking) considered. Furthermore, the MMP-8/TIMP-1 ratio correlated with TTC patients’ EFs. This association may reflect increased fibrosis and thus the left ventricle impairment severity.

Even though TnT shows high specificity for myocardial damage, the slow elevation of TnT concentrations causes problems in acute decision-making. Furthermore, other non-atherosclerotic heart conditions, like TTC, may also cause TnT elevation, making all attempts to improve the diagnostics in ACS-suspected patients worthwhile.

Collagen, especially type I, is a key load-bearing protein in the ECM of atherosclerotic plaques. Measurements both from ACS patients’ circulation and from their vulnerable atherosclerotic plaques show elevated levels of MMP-8, which seems to play a role in thinning of the atheroma-protective cap by cleaving the supportive type-I-collagen [4]. TIMP-1 has a high specificity to MMP-8; and in addition to MMP inhibition, independently exerts pro-inflammatory and growth factor-like properties [23]. TIMP-1 is also elevated in ACS patients’ serum samples [6]. Elevated MMP-8 and TIMP-1 levels, resulting from rupture of atherosclerotic plaque, thus have the potential to differentiate between ACS and TTC.

In the current study, ACS and TTC patients’ MMP-8 and TIMP-1 profiles differed. Their levels were higher in TTC and ACS patients relative to those in controls, indicating their involvement in both acute conditions. TIMP-1 differentiated ACS from TTC with higher sensitivity and specificity than did the classic myocardial damage-marker TnT. Stratification of the groups by age and by their significant clinical differences (age, sex, and smoking) enhanced this result. Conclusively, in an acute cardiac condition, low TIMP-1 and TnT levels suggest TTC rather than ACS.

The exact pathophysiology of TTC remains an open question. Thus far, the most plausible mechanisms seem to be a catecholamine surge accompanied by a change in adrenergic signalling, [24] and a microvascular dysfunction [25]. Whether their effects are mutually exclusive or synergetic is unknown [12].

Change in ECM composition is the key element of the left-ventricle remodelling found in TTC patients’ acute phase and post-recovery endomyocardial biopsies. These biopsies show a transient increase in interstitial fibrosis, composed especially of type-I collagen, in conjunction with inflammation. MMP-2, MMP-9, and TIMP-3 levels from the biopsies showed elevated acute MMP-9. The follow-up biopsies after functional recovery show ECM normalization [13,26]. Acute TTC also exhibits an increased collagen type-I/III ratio, associated with decreased elasticity and myocardial stiffness [26]. Some of the TTC patients have contraction-band necrosis similar to that in pheochromocytoma, but lack credible signs of post-ischemic necrosis [13,26].

Due to decreased proteolysis, ECM accumulates in pressure-overload such as in hypertensive heart-failure patients. Such patients have altered, pro-fibrotic, MMP and TIMP profiles. The increased amount of fibrosis in the myocardial ECM in these patients affects the left ventricle, both the form and function. Cardiac fibrosis, such as in hypertrophic cardiomyopathy, linearly associates with regional contractility [27].

Our results showed low levels of MMP-8 in conjunction with elevated TIMP-1 levels in low-EF TTC patients. The low MMP-8/TIMP-1 molar ratio may reflect reduction in proteolysis and thus increased ECM fibrosis. Considering TTC biopsy data and the previous findings in heart-failure patients, we suggest that the severity of TTC may be affected by fibrosis due to low MMP-8 and high TIMP-1 levels. Moreover, stress and neurohumoral activation, including elevated norepinephrine concentration, affects MMP levels [28,29]. Therefore, in susceptible patients, a possible catecholamine-induced disruption of the MMP-8/TIMP-1 balance may lead to a transient increase in myocardial ECM fibrosis, further impairing left-ventricle contractility. Our results showed correlation of MMP-8/TIMP-1 molar ratio with EF in TTC, further supporting the connection between fibrosis and left ventricular function.

Unlike in TTC, ACS patients’ EF did not relate to levels of TIMP-1 and MMP-8. Even though ACS patients and TTC patients with low EF had similar TIMP-1 levels, in contrast to ACS patients, those TTC patients with low EF had low MMP-8. Therefore, in differentiation between ACS and TTC, TIMP-1 appears most useful in those with less severe left ventricular impairment and possibly milder symptoms. Despite similar on-admission TnT values, the highest values during hospitalization were significantly higher in cases of ACS than of TTC. As earlier reported with MMP-1 and MMP-9 [30], the MMP-8 and TIMP-1 showed no correlation with either of the TnT values, suggesting that MMP-8 and TIMP-1 do not describe myocardial damage size. The MMP-8 and TIMP-1, however, did correlate with hsCRP, suggesting their involvement in the inflammation.

Several reports of atherosclerotic plaques and elevated serum levels of ACS patients implicate MMP-8's role in the rupture of atherosclerotic plaques. The culprit coronary lesion therefore seemed the most plausible source of MMP-8 and TIMP-1 in ACS. In TTC, the earlier evidence of transient fibrosis associated with inflammation suggested a myocardial source for the MMP-8 and TIMP-1. Our results support a differing mechanism for the MMP-8 and TIMP-1 levels in ACS and TTC. Their differing relationship to left ventricle function supports a different aetiology for the contraction abnormality. Moreover, such a relationship in TTC may offer an explanation for this varying severity of left ventricle impairment.

### Limitations

A larger number of TTC patients would have allowed for more accurate assessment of MMP-8, TIMP-1, and EF associations and would have further clarified the ability of TIMP-1 in differential diagnosis. Furthermore, no left ventriculograms of ACS patients were available, which prevented direct comparison of cardiac function and MMP-8 and TIMP-1 between ACS- and TTC patients. More direct proof of MMP-8/TIMP-1 molar ratios’ relation to ECM fibrosis would have required endomyocardial biopsies of the TTC patients.

Our study design did not allow us to discern whether the kinetics of the enzymes affected the final results. Even though the serum samples were taken in the acute phase, the time-lapse information from pain-to-blood sample was lacking. Moreover, serial sampling was impossible. Previously, serial samples of ACS patients after myocardial infarction revealed a dynamic quality in the circulating levels of MMP-8, MMP-9, and TIMP-1 in both the acute and the recovery period [31,32]. Such a finding warrants further prospective TTC studies with serial samples.

### Conclusions

Acute-phase TIMP-1 superseded other differing factors and improved the ability of TnT in differentiation between ACS and TTC. In TTC, a pro-fibrotic low MMP-8/TIMP-1 molar ratio correlated with low EF, which could reflect the previously reported transient fibrosis in endomyocardial biopsies. We suggest that the possible fibrotic accumulation of ECM might explain, in some TTC patients, their more severely impaired left ventricular function.

## Supporting information

S1 Table Odds ratios for ACS compared to TTC; TTC and ACS compared to control. (XLSX)

S2 Table TTC patients stratified by ejection fraction compared to ACS and controls. (XLSX)

S3 Table Apical vs. Mid-ventricular TTC. (XLSX)
